# Anatomical mapping of whole-brain monosynaptic inputs to the orbitofrontal cortex

**DOI:** 10.3389/fncir.2025.1567036

**Published:** 2025-04-04

**Authors:** Mei Yang, Hailing Yang, Lang Shen, Tonghui Xu

**Affiliations:** ^1^Laboratory Animal Center, Fudan University, Shanghai, China; ^2^Laboratory Animal Resource Center, Fudan University, Shanghai, China

**Keywords:** orbitofrontal cortex, neural circuit, monosynaptic tracing, whole-brain inputs, rabies virus tracing

## Abstract

The orbitofrontal cortex (ORB) exhibits a complex structure and diverse functional roles, including emotion regulation, decision-making, and reward processing. Structurally, it comprises three distinct regions: the medial part (ORBm), the ventrolateral part (ORBvl), and the lateral part (ORBl), each with unique functional attributes, such as ORBm’s involvement in reward processing, ORBvl’s regulation of depression-like behavior, and ORBl’s response to aversive stimuli. Dysregulation of the ORB has been implicated in various psychiatric disorders. However, the neurocircuitry underlying the functions and dysfunctions of the ORB remains poorly understood. This study employed recombinant adeno-associated viruses (rAAV) and rabies viruses with glycoprotein deletion (RV-ΔG) to retrogradely trace monosynaptic inputs to three ORB subregions in male C57BL/6J mice. Inputs were quantified across the whole brain using fluorescence imaging and statistical analysis. Results revealed distinct input patterns for each ORB subregion, with significant contributions from the isocortex and thalamus. The ORBm received prominent inputs from the prelimbic area, agranular insular area, and hippocampal field CA1, while the ORBvl received substantial intra-ORB inputs. The ORBl exhibited strong inputs from the somatomotor and somatosensory areas. Thalamic inputs, particularly from the mediodorsal nucleus and submedial nucleus of the thalamus, were widespread across all ORB subregions. These findings provide novel insights into the functional connectivity of ORB subregions and their roles in neural circuit mechanisms underlying behavior and psychiatric disorders.

## Introduction

The orbitofrontal cortex (ORB) is situated near the eye socket, in the antero-inferior section of the frontal lobe. It plays a vital role in rewards processing, emotional regulation, and decision-making (Yang et al., 2019; Padoa-Schioppa and Assad, 2006; Salzman and Fusi, 2010; Rolls, 2023). It comprises three distinct subregions: the medial (ORBm), ventrolateral (ORBvl), and lateral (ORBl) parts, each with unique contributions. Research has revealed functional heterogeneity between the ORBm and ORBl in decision-making and visual reversal learning. For instance, rats with ORBm lesions show reduced impulsivity, while rats with ORBl lesions show increased impulsivity (Mar et al., 2011). Inactivation of the ORBl impairs serial visual reversal learning due to perseveration errors, whereas ORBm inactivation enhances it by reducing perseveration (Hervig et al., 2020). Each subregion of the ORB demonstrates unique functions. The ORBm is typically involved in anticipating and evaluating rewards (Critchley and Rolls, 1996; Padoa-Schioppa and Assad, 2006), whereas the ORBl plays a crucial role in processing punishment during rewards-related tasks, with the ORBI’s activation level mirroring punishment severity (O’Doherty et al., 2001). When faced with multiple options, the ORBvl contributes to precise decision-making by relaying information to the dorsomedial striatum (Gore et al., 2023).

The ORB fulfills a range of intricate and diverse functions, and its dysregulation has been directly linked to the onset of various psychiatric disorders, such as major depression (Baxter et al., 1987; Sheng et al., 2020; Zhao et al., 2017), schizophrenia (Núñez et al., 2024), parkinsonism (du Plessis et al., 2018), and obsessive-compulsive disorder (OCD) (Beucke et al., 2013). For example, individuals with schizophrenia show a marked decrease in regional cerebral blood flow within the right ORB (Kanahara et al., 2013). Patients with parkinsonism exhibit impaired ORB activation during both rewards anticipation and outcome processing (du Plessis et al., 2018). The connection between OCD and ORB abnormalities has also become increasingly clear. Patients with OCD have been shown to have reduced ORB volumes and higher ORB metabolic rates (Choi et al., 2004; Baxter et al., 1987). Collectively, these findings underscore a strong correlation between ORB abnormalities and the emergence and progression of some disorders, suggesting that the ORB may be a promising target for treatment.

Given the complexity and significance of ORB function, its neural connections within the brain have been the focus of numerous studies. Rodent research has extensively explored ORB projections using various tracing techniques, revealing widespread targets excluding the hippocampus and cerebellum. These include the primary olfactory cortex (Babalian et al., 2019), piriform cortex (Illig, 2005), caudate putamen (Schilman et al., 2008), amygdala (McDonald et al., 1996), extended amygdala (Reynolds and Zahm, 2005), submedial thalamic nucleus (SMT) and mediodorsal thalamic nuclei (MD) (Yoshida et al., 1992; Reep et al., 1996), parafascicular nucleus (Jones and Leavitt, 1974), claustrum (Zhang et al., 2001), lateral hypothalamus (Hardy, 1994), periaqueductal gray (Hardy, 1986; Babalian et al., 2019), and oculomotor complex (Leichnetz et al., 1987). Concurrently, the ORB integrates diverse inputs from sensory, limbic, and thalamic regions. Anterograde tracing has shown rostral basolateral amygdala nucleus (BLA) projections to ORBl (Kita and Kitai, 1990), while retrograde studies have highlighted subregion-specific thalamocortical connectivity: ORBvl receives dense inputs from the insular cortex and somatosensory areas, whereas ORBm prefers connectivity with the cingulate and posterior parietal cortices. Thalamic afferents are organized subregion-specifically, with ORBvl innervated by the SMT and ORBm/ORBl by the periphery of SMT and specific MD segments (Reep et al., 1996). Recent rodent studies have identified novel pathways, such as postrhinal cortex inputs to ORBvl and reciprocal connectivity between the lateral agranular part of the retrosplenial area (RSPagl) and ORBm (Qi et al., 2019; Xiang et al., 2023). Insular cortex projections preferentially target medial and lateral ORB, sparing ventral subregions (Mathiasen et al., 2023).

In recent years, research on ORB neural circuits has deepened. In 2021, the retrograde tracer cholera toxin subunit B was used to label projections into different ORB subregions, revealing distinct projection patterns from various brain regions to the anterior lateral, posterior lateral, and posterior ventral portions of the ORB (Barreiros et al., 2021). The same year, combining neuronal sparse and bright labeling with whole-brain fluorescence imaging yielded an uninterrupted 3D whole-brain dataset, enabling the morphological reconstruction of 25 ORB pyramidal neurons and revealing divergent projection patterns for information delivery across cortical layers (Wang et al., 2021). In 2022, efferent projections from the five ORB divisions to the thalamus (TH) were studied, demonstrating distinct projection patterns for each division (Vertes et al., 2023). In 2023, inputs to five types of projection-specific ORB neurons and outputs to two types of inhibitory neurons were mapped, revealing that similar cortical and thalamic regions innervate different projection-defined ORB neurons (Zhang et al., 2024).

However, many of these studies, particularly input studies, have relied on traditional tracing strategies that cannot achieve monosynaptic retrograde cellular-level labeling across single synapses like RV. Additionally, they often lack ORB subregion labeling and quantitative brain-wide comparisons. Therefore, our objective is to conduct a quantitative analysis and comparison of whole-brain inputs to various ORB subregions, delineating their similarities and differences and providing new insights into the neural circuit mechanisms underlying their functional characteristics and correlations.

In this study, we employed recombinant adeno-associated viruses (rAAV) and rabies viruses with glycoprotein deletion (RV-ΔG) to achieve retrograde trans-monosynaptic labeling of the ORBm, ORBvl, and ORBl. Fluorescence imaging and detailed quantitative analysis yielded significant insights. Specifically, the ORB subregions receive considerable inputs from both the cerebral cortex (CTX) and TH, with each subregion displaying a distinct pattern of preferential inputs from specific brain areas. These unique input profiles underscore the diverse functional connectivity of the ORB and hint at their potential specialized roles in processing and integrating information within the brain’s complex neural networks.

## Materials and methods

### Animals

C57BL/6J male mice (8–12 weeks old, *n* = 12) were used in this study. These mice were housed under standardized conditions (12 h of light/12 h of darkness, temperature: 21–24°C, humidity: 50%–60%), with free access to food and water. All animal experiments were approved by the Laboratory Animal Center, Fudan University.

### Virus injection

The mice were fixed in a stereotaxic apparatus (RWD, China) under anesthesia with isoflurane. All viral tools used in this study were packaged and provided by Brain VTA (China). We mixed the rAAV-hSyn-RG-WPRE-hGB-polyA (titer: 5.47E + 12 vg/ml) and rAAV-hSyn-His-EGFP-2a-TVA-WPRE (titer: 3.21E + 12 vg/ml) in a ratio of 1:1. After drilling a hole in the skull above the target ORB subregion of the right brain, 10 nL of mixed AAV viruses was injected into the target ORB subregion (ORBm, A/P: 2.40 mm, M/L: –0.20 mm, D/V: –2.50 mm; ORBvl, A/P: 2.40 mm, M/L: –0.75 mm, D/V: –2.50 mm; ORBl, A/P: 2.50 mm, M/L: –1.45 mm, D/V: –2.50 mm). A total of 2 weeks later, the same coordinate was injected with 150 nL of RV-CVS-EnvA-N2C(ΔG)-tdTomato (titer: 2.00E + 08 IFU/ml). The virus injection was performed at a rate of 10 nL/min. After the injection, the pipette was held for 10 min before being slowly retracted from the brain to prevent backflow of the virus.

### Slice preparation

A total of 1 week after the RV virus injection, mice were perfused transcardially with 0.01 M phosphate-buffered saline (PBS), followed by 4% paraformaldehyde (PFA). The perfused brain was removed from the skull and stored in 4% PFA at 4°C overnight. Brains were then embedded with agarose. In our study, the entire mouse brain was sectioned coronally, spanning from +6.0 mm anterior to –8.5 mm posterior relative to bregma, to ensure comprehensive coverage of all potential input regions. Serial sections (50 μm thickness) were cut with VT1200S vibratome (Leica Biosystems, Germany), and every second section was systematically selected for imaging and quantitative analysis to balance resolution with sampling efficiency. All sections were coverslipped using glycerol.

### Image and data analysis

For fluorescence imaging, we acquired overview images of the brain with a 10× objective on a fluorescence scanner (Nikon Ni-E, Japan) or high-magnification images on a confocal microscope (Olympus FV3000, Japan). Imaging utilized two channels: the 575 nm laser line was used to image the tdTomato channel for cells located outside the injection site, and the 488 nm and 575 nm laser lines were used to image the EGFP and tdTomato channels for cells at the injection site. The image post-processing was conducted using ImageJ, Photoshop, and GraphPad Prism software.

### Statistical analysis

We counted the number of cells in each brain region, and excluded cells only showing red in the target ORB subregion. Brain regions are divided according to the Allen Mouse Brain Atlas (Wang Q. et al., 2020). All related subregions and their abbreviations are listed in Table 1. The statistical analysis was performed in GraphPad Prism (United States). One-way repeated measures ANOVA and Student’s *t*-test were used to determine the significance of the effect. Data were expressed as the Mean ± SEM. *P* < 0.05 was considered to be statistically significant. *P < 0.05, **P < 0.01, ***P < 0.001.

**TABLE 1:** Abbreviations.

Abbreviation	Definition
ACA	Anterior Cingulate Area
ACAd	Anterior cingulate area, dorsal part
ACAv	Anterior cingulate area, ventral part
AI	Agranular insular area
AId	Agranular insular area, dorsal part
AIp	Agranular insular area, posterior part
AIv	Agranular insular area, ventral part
AM	Anteromedial nucleus
AM	Anteromedial nucleus
AON	Anterior olfactory nucleus
ATN	Anterior group of the dorsal thalamus
AUD	Auditory Areas
BLA	Basolateral amygdalar nucleus
BLAa	Basolateral amygdalar nucleus, anterior part
BLAp	Basolateral amygdalar nucleus, posterior part
BLAv	Basolateral amygdalar nucleus, ventral part
BMA	Basomedial amygdalar nucleus
CA	Ammon’s horn
CA1	Field CA1
CB	Cerebellum
CL	Central lateral nucleus of the thalamus
CLA	Claustrum
CM	Central medial nucleus of the thalamus
COA	Cortical amygdalar area
CTX	Cerebral cortex
CTXsp	Cortical subplate
ECT	Ectorhinal area
EGFP	Enhanced green fluorescent protein
ENT	Entorhinal area
ENTl	Entorhinal area, lateral part
EnvA	Envelope protein of the avian sarcoma and leukosis virus
EP	Endopiriform nucleus
EPI	Epithalamus
FRP	Frontal Pole
GENd	Geniculate group, dorsal thalamus
GENv	Geniculate group, ventral thalamus
GU	Gustatory areas
HB	Hindbrain
HIP	Hippocampal region
HPF	Hippocampal formation
HY	Hypothalamus
IAD	Interanterodorsal nucleus of the thalamus
ILA	Infralimbic Area
ILM	Intralaminar nuclei of the dorsal thalamus
IPN	Interpeduncular nucleus
LA	Lateral amygdalar nucleus
LAT	Lateral group of the dorsal thalamus
LHA	Lateral hypothalamic area
LP	Lateral posterior nucleus of the thalamus
MB	Midbrain
MD	Mediodorsal nucleus of thalamus
MED	Medial group of the dorsal thalamus
MO	Somatomotor areas
MOB	Main olfactory bulb
MOp	Primary motor area
MOs	Secondary motor area
MTN	Midline group of the dorsal thalamus
NLOT	Nucleus of the lateral olfactory tract
OCD	Obsessive-compulsive disorder
OLF	Olfactory areas
ORB	Orbitofrontal cortex
ORBl	Orbitofrontal cortex, lateral part
ORBm	Orbitofrontal cortex, medial part
ORBvl	Orbitofrontal cortex, ventrolateral part
PA	Posterior amygdalar nucleus
PAA	Piriform-amygdalar area
PAG	Periaqueductal gray
PAL	Pallidum
PB	Parabrachial nucleus
PBS	Phosphate buffered solution
PCN	Paracentral nucleus
PDTg	Posterodorsal tegmental nucleus
PERI	Perirhinal area
PF	Parafascicular nucleus
PFA	Paraformaldehyde
PIR	Piriform area
PL	Prelimbic Area
PO	Posterior complex of the thalamus
PPN	Pedunculopontine nucleus
PR	Perireunensis nucleus
PT	Parataenial nucleus
PTLp	Posterior parietal association areas
PVT	Paraventricular nucleus of the thalamus
rAAV	Recombinant adeno-associated viruses
RE	Nucleus of reuniens
RG	Rabies glycoprotein
RH	Rhomboid nucleus
RHP	Retrohippocampal region
RSP	Retrosplenial area
RSPagl	Retrosplenial area, lateral agranular part
RSPd	Retrosplenial area, dorsal part
RSPv	Retrosplenial area, ventral part
RT	Reticular nucleus of the thalamus
RV-ΔG	Rabies Virus with Glycoprotein Deletion
SMT	Submedial nucleus of the thalamus
SNr	Substantia nigra, reticular part
SPA	Subparafascicular area
SPF	Subparafascicular nucleus
SS	Somatosensory areas
SSp	Primary somatosensory area
SSs	Supplemental somatosensory area
STR	Striatum
TEa	Temporal association areas
TH	Thalamus
TT	Taenia tecta
VAL	Ventral anterior-lateral complex of the thalamus
VENT	Ventral group of the dorsal thalamus
VIS	Visual areas
VISC	Visceral area
VM	Ventral medial nucleus of the thalamus
VTA	Ventral tegmental area

## Results

### Tracing the monosynaptic inputs to the ORB

**FIGURE 1:**
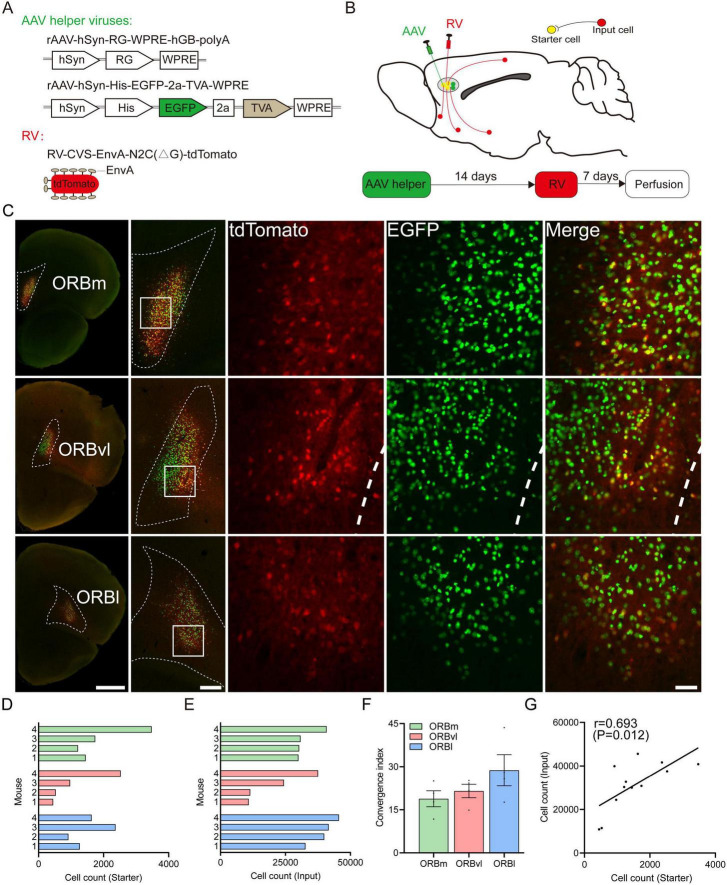
Experimental strategy for identifying monosynaptic inputs to three the orbitofrontal cortex (ORB) subregions. **(A)** Viral vector design of recombinant adeno-associated viruses (rAAV) and rabies viruses with glycoprotein deletion (RV-ΔG) viruses. **(B)** Schematic illustration of retrograde trans-synaptic tracing and the experimental timeline. **(C)** Representative coronal brain sections showing starter cells in the ORB subregions. Scale bars, from left to right, 1,000, 250, 50 μm. **(D)** Number of starter cells in the medial part (ORBm), the ventrolateral part (ORBvl), and the lateral part (ORBl), respectively (*n* = 4 mice/group). **(E)** Number of input cells in the ORB subregions (*n* = 4 mice/group). **(F)** Ratio between the number of input cells and starter cells. Data are presented as the Mean ± SEM (*n* = 4 mice/group). **(G)** Linear relationship between the number of starter cells and input cells. For abbreviations, see Table 1.

Our primary objective was to identify the neurons that participate in monosynaptic upstream connections with distinct subregions of the ORB. We employed rAAV-hSyn-RG-WPRE-hGB-polyA (AAV-RG), rAAV-hSyn-His-EGFP-2a-TVA-WPRE (AAV-EGFP-TVA), and RV-CVS-EnvA-N2C(ΔG)-tdTomato (RV-ΔG) vectors to map these connections (Figure 1A). RV-ΔG, a pivotal retrograde tracer, plays a crucial role in delineating neural circuits across monosynaptic connections (Ohara et al., 2013; Gehrlach et al., 2020; Watabe-Uchida et al., 2012; Masaki et al., 2022). This virus initiates an interaction between the envelope protein of the avian sarcoma and leukosis virus (EnvA) and the TVA receptor to infect cells, subsequently traversing synaptic junctions through the utilization of the rabies glycoprotein (RG). We initially injected two AAV viruses into the target ORB subregion. One AAV expressed TVA, allowing RV-ΔG to recognize and infect cells, while the other encoded the RG, enabling RV-ΔG to traverse a single synapse. Following a 2 weeks incubation period, we administered RV-ΔG into the same ORB subregion, which allowed us to identify the upstream neurons that participated in monosynaptic connections with each ORB subregion (Figure 1B). This approach enabled us to identify and trace the neural pathways underlying the complex connectivity of the ORB subregions.

At the injection site, cells infected with AAV-EGFP-TVA served a dual purpose: they expressed 1) TVA to enable subsequent infection by RV-ΔG and 2) enhanced green fluorescent protein (EGFP), which was a reliable marker for identifying TVA-expressing cells and assessing injection accuracy. The RV-ΔG vector also expressed the red fluorescent protein, tdTomato, which enabled the tracing of upstream neuronal connections. When neurons were co-infected with AAV-EGFP-TVA and RV-ΔG, they exhibited yellow fluorescence due to the concurrent expression of EGFP and tdTomato. As such, they were identified as starter cells. Within these starter cells, the RV-ΔG virus continued to replicate and spread in a retrograde trans-synaptic manner, which labeled upstream neurons with red fluorescence. These upstream neurons were identified as input cells (Figure 1B). Notably, our experiments demonstrated that over 85% of starter cells were confined to their target regions (Figure 1C), demonstrating the precision and efficacy of our viral injection technique.

We conducted qualitative and quantitative analyses of these input cells to identify the whole-brain input patterns of the ORB subregions. We started by counting the number of starter cells within each ORB subregion, respectively (Figures 1D, E). The ratios of input cells to starter cells varied significantly across the subregions (ORBm: 18.80 ± 2.79, ORBvl: 21.50 ± 2.33, ORBl: 28.75 ± 5.44, Figure 1F). However, a robust and consistent correlation was observed between the number of input cells and starter cells of all animal samples (Figure 1G), validating the reliability of our data despite sample variations.

### Overview of whole-brain inputs to the ORB

**FIGURE 2:**
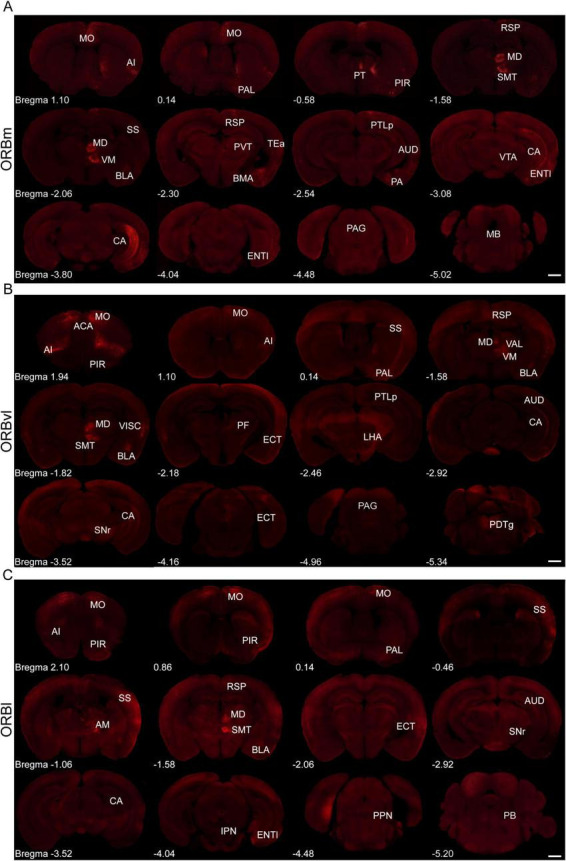
Representative fluorescence images depicting input cells within whole-brain regions for the medial part (ORBm), the ventrolateral part (ORBvl), and the lateral part (ORBl) groups. **(A–C)** Representative fluorescence images of the ORBm, ORBvl, and ORBl groups (Scale bar, 1,000 μm).

We analyzed the brain-wide input distribution to the ORB subregions by quantifying the input intensity originating from the ipsilateral and contralateral brain areas vis-a-vis our injection sites. The ORB subregions received strong inputs from multiple brain regions, with the majority originating from the ipsilateral side (Figure 2). To validate the precision of viral targeting, representative EGFP fluorescence images of the injection sites for all three samples shown in Figure 2 are provided in Supplementary Figure 1. Specifically, the ipsilateral side provided 76.96 ± 0.96%, 75.24 ± 3.74%, and 67.50 ± 3.68% of the total inputs to the ORBm, ORBvl, and ORBl, respectively. The input neurons received by ORBm, ORBvl, and ORBl are not randomly distributed. They are primarily located in the ipsilateral cerebral cortex and TH region, while the contralateral input neurons are predominantly distributed in the contralateral ORB (Supplementary Figure 2).

**FIGURE 3:**
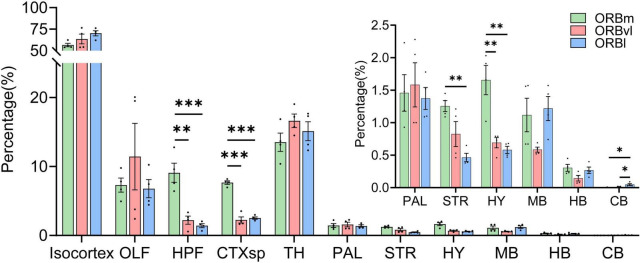
Distribution of input cells projecting to the orbitofrontal cortex (ORB) subregions. *P < 0.05, **P < 0.01, ***P < 0.001. Data are presented as the Mean ± SEM (*n* = 4 mice/group). One-way ANOVA combined with Tukey’s multiple comparisons test.

The ORB received (Table 2) inputs of varying intensities from 167 distinct brain regions, which can be grouped into 11 major areas: the isocortex, olfactory areas (OLF), hippocampal formation (HPF), cortical subplate (CTXsp), TH, pallidum (PAL), striatum (STR), hypothalamus (HY), midbrain (MB), hindbrain (HB), and cerebellum (CB) (Figure 3). The input patterns observed in the ORB subregions exhibited certain similarities. The isocortex emerged as the predominant input region, accounting for over half of the total inputs. The isocortex was followed by the TH, which contributed approximately 15%. There were relatively fewer inputs from the PAL, STR, HY, and MB, all with comparable intensity. The three ORB subregions also exhibited input patterns that were significantly different from one another. For example, the ORBm received considerably more inputs from the HPF, CTXsp, STR, and HY compared to the ORBvl or ORBl (Figure 3, *P* < 0.01). Since the primary inputs to the ORB originated from the CTX (through the isocortex, OLF, HPF, and CTXsp), and TH, we subsequently focused our analysis on these brain regions.

**TABLE 2:** The list of 167 brain regions projecting to the orbitofrontal cortex (ORB).

Abbreviation	Definition
AAA	Anterior amygdalar area
ACA	Anterior cingulate area
ACB	Nucleus accumbens
AD	Anterodorsal nucleus
ADP	Anterodorsal preoptic nucleus
AHN	Anterior hypothalamic nucleus
AI	Agranular insular area
AM	Anteromedial nucleus
AON	Anterior olfactory nucleus
APN	Anterior pretectal nucleus
ARH	Arcuate hypothalamic nucleus
AT	Anterior tegmental nucleus
AUD	Auditory areas
AV	Anteroventral nucleus of thalamus
AVP	Anteroventral preoptic nucleus
AVPV	Anteroventral periventricular nucleus
BLA	Basolateral amygdalar nucleus
BMA	Basomedial amygdalar nucleus
BST	Bed nuclei of the stria terminalis
CA	Ammon’s horn
CEA	Central amygdalar nucleus
CL	Central lateral nucleus of the thalamus
CLA	Claustrum
CM	Central medial nucleus of the thalamus
COA	Cortical amygdalar area
CP	Caudoputamen
CS	Superior central nucleus raphe
CUN	Cuneiform nucleus
DMH	Dorsomedial nucleus of the hypothalamus
DN	Dentate nucleus
DP	Dorsal peduncular area
DR	Dorsal nucleus raphe
ECT	Ectorhinal area
ENT	Entorhinal area
EP	Endopiriform nucleus
Eth	Ethmoid nucleus of the thalamus
FN	Fastigial nucleus
FRP	Frontal pole, cerebral cortex
FS	Fundus of striatum
Gpe	Globus pallidus, external segment
GPi	Globus pallidus, internal segment
GRN	Gigantocellular reticular nucleus
GU	Gustatory areas
HATA	Hippocampo-amygdalar transition area
IA	Intercalated amygdalar nucleus
IAD	Interanterodorsal nucleus of the thalamus
IAM	Interanteromedial nucleus of the thalamus
IF	Interfascicular nucleus raphe
ILA	Infralimbic area
IMD	Intermediodorsal nucleus of the thalamus
INC	Interstitial nucleus of Cajal
IP	Interposed nucleus
IPN	Interpeduncular nucleus
IRN	Intermediate reticular nucleus
KF	Koelliker-Fuse subnucleus
LA	Lateral amygdalar nucleus
LD	Lateral dorsal nucleus of thalamus
LDT	Laterodorsal tegmental nucleus
LGd	Dorsal part of the lateral geniculate complex
LGv	Ventral part of the lateral geniculate complex
LH	Lateral habenula
LHA	Lateral hypothalamic area
LM	Lateral mammillary nucleus
LP	Lateral posterior nucleus of the thalamus
LPO	Lateral preoptic area
LS	Lateral septal nucleus
MA	Magnocellular nucleus
MARN	Magnocellular reticular nucleus
MD	Mediodorsal nucleus of thalamus
MEA	Medial amygdalar nucleus
MG(m)	Medial geniculate complex, medial part
MH	Medial habenula
MM	Medial mammillary nucleus
MO	Somatomotor areas
MOB	Main olfactory bulb
MPN	Medial preoptic nucleus
MPO	Medial preoptic area
MRN	Midbrain reticular nucleus
MS	Medial septal nucleus
MT	Medial terminal nucleus of the accessory optic tract
MV	Medial vestibular nucleus
NDB	Diagonal band nucleus
NI	Nucleus incertus
NLOT	Nucleus of the lateral olfactory tract
NPC	Nucleus of the posterior commissure
OLF	Olfactory areas
ORB	Orbital area
OT	Olfactory tubercle
PA	Posterior amygdalar nucleus
PAA	Piriform-amygdalar area
PAG	Periaqueductal gray
PARN	Parvicellular reticular nucleus
PB	Parabrachial nucleus
PCG	Pontine central gray
PCG	Pontine central gray
PCN	Paracentral nucleus
PeF	Perifornical nucleus
PERI	Perirhinal area
PF	Parafascicular nucleus
PH	Posterior hypothalamic nucleus
PIL	Posterior intralaminar thalamic nucleus
PIR	Piriform area
PL	Prelimbic area
PM	Premammillary nucleus
PO	Posterior complex of the thalamus
POL	Posterior limiting nucleus of the thalamus
PoT	Posterior triangular thalamic nucleus
PPN	Pedunculopontine nucleus
PR	Perireunensis nucleus
PRC	Precommissural nucleus
PRE	Presubiculum
PRNr	Pontine reticular nucleus
ProS	Prosubiculum
PS	Parastrial nucleus
PSTN	Parasubthalamic nucleus
PSV	Principal sensory nucleus of the trigeminal
PT	Parataenial nucleus
PTLp	Posterior parietal association areas
PVH	Paraventricular hypothalamic nucleus
PVT	Paraventricular nucleus of the thalamus
RCH	Retrochiasmatic area
RE	Nucleus of reuniens
RH	Rhomboid nucleus
RL	Rostral linear nucleus raphe
RM	Nucleus raphe magnus
RN	Red nucleus
RPF	Retroparafascicular nucleus
RPO	Nucleus raphe pontis
RR	Midbrain reticular nucleus, retrorubral area
RSP	Retrosplenial area
RT	Reticular nucleus of the thalamus
SCm	Superior colliculus, motor related
SF	Septofimbrial nucleus
SGN	Suprageniculate nucleus
SI	Substantia innominata
SMT	Submedial nucleus of the thalamus
SNc	Substantia nigra, compact part
SNr	Substantia nigra, reticular part
SO	Supraoptic nucleus
SOC	Superior olivary complex
SPA	Subparafascicular area
SPF	Subparafascicular nucleus
SPVO	Spinal nucleus of the trigeminal, oral part
SS	Somatosensory areas
STN	Subthalamic nucleus
SUB	Subiculum
SUM	Supramammillary nucleus
SUT	Supratrigeminal nucleus
TEa	Temporal association areas
TMv	Tuberomammillary nucleus, ventral part
TR	Postpiriform transition area
TRN	Tegmental reticular nucleus
TRS	Triangular nucleus of septum
TT	Taenia tecta
TU	Tuberal nucleus
VAL	Ventral anterior-lateral complex of the thalamus
VIS	Visual areas
VISC	Visceral area
VLPO	Ventrolateral preoptic nucleus
VM	Ventral medial nucleus of the thalamus
VMH	Ventromedial hypothalamic nucleus
VPL	Ventral posterolateral nucleus of the thalamus
VPM	Ventral posteromedial nucleus of the thalamus
VTA	Ventral tegmental area
VTN	Ventral tegmental nucleus
Xi	Xiphoid thalamic nucleus
ZI	Zona incerta

### Inputs from the CTX to the ORB

**FIGURE 4:**
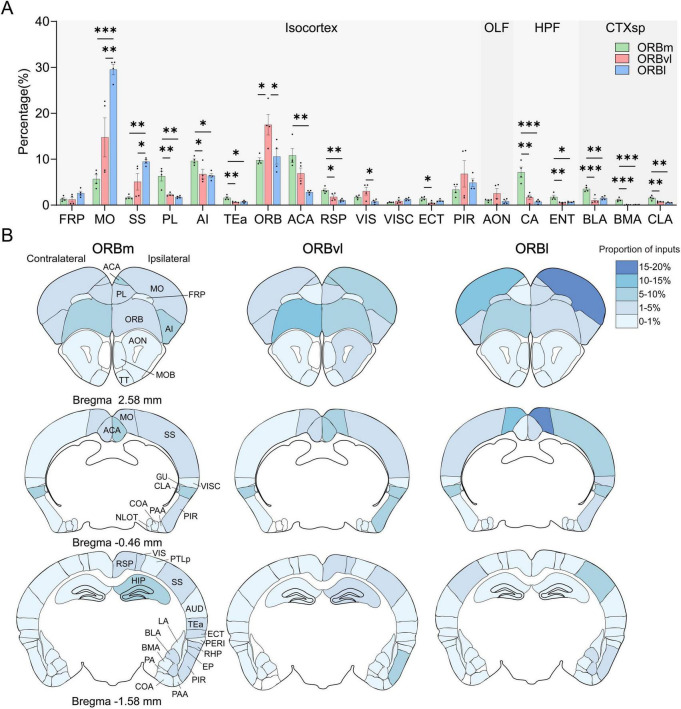
Cerebral cortex (CTX) inputs to the the orbitofrontal cortex (ORB) subregions. **(A)** Distribution of input cells projecting to the ORB subregions in the CTX. **(B)** Mean proportion of inputs from different CTX regions to the ORB. The input ratio shown in white is 0%. *P < 0.05, **P < 0.01, ***P < 0.001. Data are presented as the Mean ± SEM (*n* = 4 mice/group). One-way ANOVA combined with Tukey’s multiple comparisons test.

We identified 38 regions within the CTX that projected into the ORB. Seventeen of these regions originated from the isocortex, whereas nine, six, and six originated from the OLF, HPF, and CTXsp, respectively. To increase the accuracy and precision of our study, we focused on the 19 cortical regions that each provided > 1% of the input to the ORB subregions (Figure 4A).

Distinct cortical brain regions demonstrated preferential connectivity patterns with the ORB. For example, the prelimbic area (PL), agranular insular area (AI), retrosplenial area (RSP), ammon’s horn (CA), and BLA were significantly more likely to provide inputs to the ORBm compared to the ORBvl or ORBl (Figures 4A, *P* < 0.05). There were also consistent inputs observed from the MO and SS to the ORB subregions, with the highest input directed toward the ORBl, followed by the ORBvl and ORBm (Figures 4A, *P* < 0.05). The ORB itself provided more inputs toward the ORBvl (Figure 4A, *P* < 0.05).

We subsequently computed for the number of ipsilateral and contralateral input cells relative to the total number of input cells. Figure 4B illustrates the intensity distribution of inputs to the ORB subregions across the entire brain. The majority of inputs originated from ipsilateral areas, whereas contralateral inputs were predominantly observed in the MO and ORB. Notably, the proportion of inputs from the contralateral ORB to the three ORB subregions surpassed that from the ipsilateral ORB to the same subregions (contralateral ORB-ORBm, ORBvl, ORBl: 6.94 ± 0.28%, 12.47 ± 1.26%, 9.59 ± 1.65%, ipsilateral ORB-ORBm, ORBvl, ORBl: 2.84 ± 0.25%, 5.04 ± 1.01%, 1.00 ± 0.18%, Supplementary Figure 3).

**FIGURE 5:**
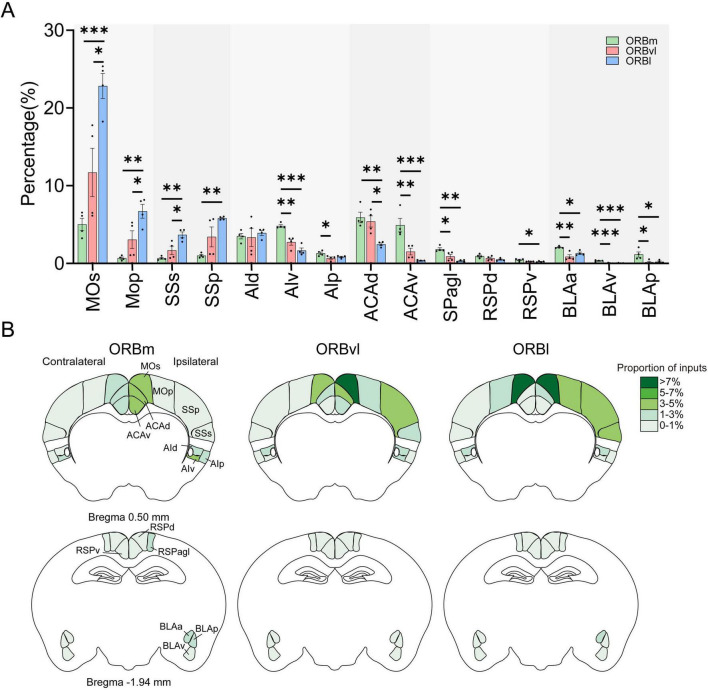
Input cells to the orbitofrontal cortex (ORB) subregions from the MO, SS, AI, ACA, RSP, and BLA. **(A)** Distribution of input cells projecting to the ORB subregions from the MO, SS, AI, ACA, RSP, and BLA. **(B)** Mean proportion of inputs to the ORB subregions from the MO, SS, AI, ACA, RSP, and BLA. *P < 0.05, **P < 0.01, ***P < 0.001. Data are represented by the Mean ± SEM (*n* = 4 mice/group). One-way repeated measures ANOVA followed by Tukey’s multiple comparisons test.

The MO, SS, AI, anterior cingulate area (ACA), RSP, and BLA preferentially provided inputs to the ORB (Figure 5A). Specifically, the primary (MOp), secondary motor areas (MOs), primary (SSp), and supplemental somatosensory areas (SSs) exhibited comparable input tendencies, with the most inputs directed toward the ORBl, followed by the ORBvl and ORBm (Figure 5A). Conversely, the ventral part of the agranular insular area (AIv), the dorsal part of the anterior cingulate area (ACAd), the ventral part of the anterior cingulate area (ACAv), the RSPagl, and the anterior part of the basolateral amygdalar nucleus (BLAa) provided inputs primarily to the ORBm, followed by the ORBvl and ORBl (Figure 5A, *P* < 0.05). Figure 5B illustrates the bilateral input patterns of these brain subregions to the ORB. There is a notable predominance of the ipsilateral hemisphere (Supplementary Figure 4).

We also conducted a quantitative analysis of the input strength of distinct cortical regions within their respective subregions to the ORB. Some brain subregions exhibited consistent input patterns to the three ORB subregions. For instance, the MOs, ACAd, and BLAa exhibited more intense inputs to each ORB subregion compared to other brain subregions within their respective regions. All other brain subregions exhibited various input patterns to the ORB subregions. For example, the AIv provided the most inputs to the ORBm, followed by the dorsal (AId) and posterior (AIp) parts of the AI. In comparison, the AId provided the most inputs to the ORBl, followed by the AIv and AIp (Supplementary Figure 5).

To further delineate hippocampal inputs to the ORB subregions, we quantified contributions from specific HPF subregions. Over 90% of HPF-derived inputs originated from the Field CA1 (CA1) (ORBm: 77.16 ± 2.65%, ORBvl: 66.16 ± 5.65%, ORBl:46.72 ± 9.16%) and lateral part of the entorhinal area (ENTl) (ORBm: 19.43 ± 2.86%, ORBvl: 28.05 ± 7.24%, ORBl:51.82 ± 8.61%), whereas inputs from Field CA2 (ORBm: 0.67 ± 0.28%, ORBvl: 2.46 ± 2.34%, ORBl:0.38 ± 0.38%), Field CA3 (ORBm: 1.06 ± 0.16%, ORBvl: 2.53 ± 2.40%, ORBl:0.38 ± 0.38%) and the subiculum (SUB) (ORBm: 0.36 ± 0.12%, ORBvl: 0.46 ± 0.24%, ORBl:0.12 ± 0.12%) were minimal (Supplementary Figure 6). Additionally, CA1 and ENTl provide preferential input to ORBm.

### Intra-ORB connectivity: inputs from ORB subregions to other ORB subregions

**FIGURE 6:**
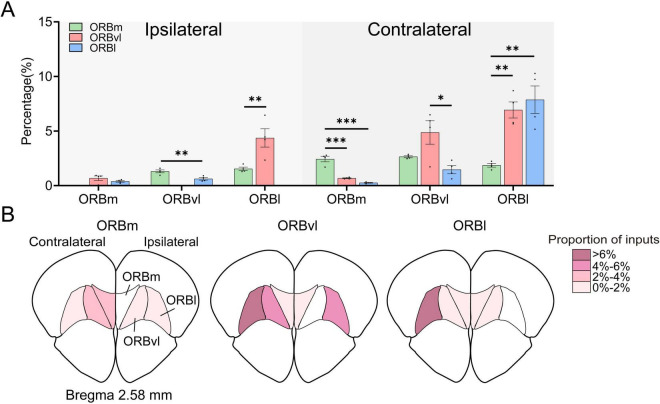
Local inputs to the orbitofrontal cortex (ORB) subregions. **(A)** Distribution of input cells projecting to the ORB subregions from the ipsilateral and contralateral ORB subregions. The horizontal coordinate represents the input brain subregion. **(B)** Mean proportion of inputs within the ORB subregions. The total number of input cells calculated for each subregion excluded the input cells native to the target ORB subregion for injection administration. *P < 0.05, **P < 0.01, ***P < 0.001. Data are presented as the Mean ± SEM (*n* = 4 mice/group). One-way ANOVA combined with Tukey’s multiple comparisons test.

Our results demonstrated the presence of strong intra-ORB projections. Strikingly, each subregion received more inputs from the contralateral hemisphere than the ipsilateral hemisphere. We conducted a more detailed analysis of these internal projections but excluded the input cells originating from each target subregion. The ORB subregions received significant inputs from bilateral ORB, with the ORBm, ORBvl, and ORBl receiving 9.78 ± 0.53%, 17.51 ± 2.24%, and 10.60 ± 1.66%, respectively. The ipsilateral ORBvl sent more projections to the ORBm than ORBl, whereas the ipsilateral ORBl sent more projections to the ORBvl than ORBm (Figure 6A, *P* < 0.01). Notably, the contralateral ORB subregions provided the most input to their respective ORB subregions, with the contralateral ORBm, ORBvl, and ORBl providing the largest inputs to the ORBm, ORBvl, and ORBl, respectively (Figure 6A, *P* < 0.01). These findings suggest that ORB subregions receive strong inputs from their contralateral counterparts, implying that the ORB may regulate its activity through internal connections. Figure 6B provides an intuitive illustration of the connectivity strengths among the various subregions within the ORB.

### Inputs from the TH to the ORB

**FIGURE 7:**
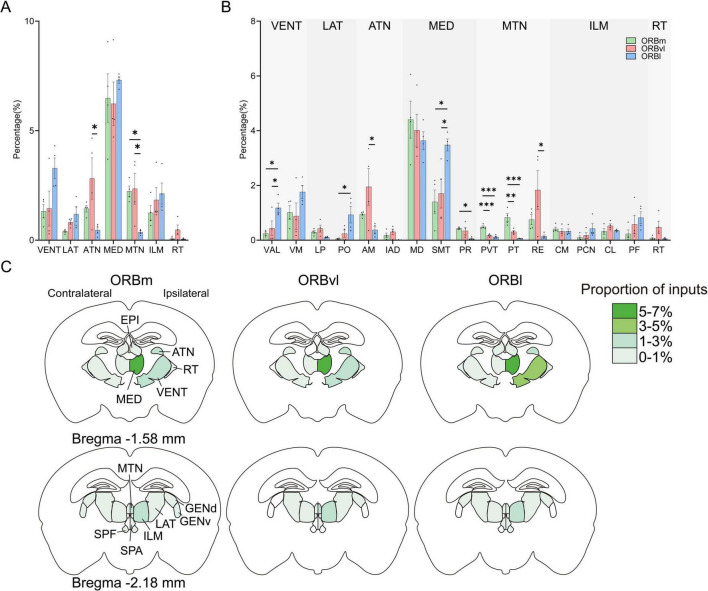
Thalamic inputs to the orbitofrontal cortex (ORB) subregions. **(A)** Distribution of input cells from the thalamus (TH) to the ORB subregions. **(B)** Distribution of input cells from the TH to the ORB subregions at the finer level of organization. **(C)** Mean proportion of inputs to the ORB subregions within the different thalamic regions. *P < 0.05, **P < 0.01, ***P < 0.001. Data are presented as the Mean ± SEM (*n* = 4 mice/group). One-way ANOVA combined with Tukey’s multiple comparisons test.

The ORB subregions received substantial inputs from the CTX and TH. The TH is segmented into 13 distinct regions, seven of which obviously participate in ORB signaling: (1) the ventral group of the dorsal TH (VENT), (2) lateral group of the dorsal TH (LAT), (3) anterior group of the dorsal TH (ATN), (4) medial group of the dorsal TH (MED), (5) midline group of the dorsal TH (MTN), (6) intralaminar nuclei of the dorsal TH (ILM), and (7) the reticular nucleus of the TH (RT) (Figure 7A). A deeper analysis of these seven regions revealed that the MED’s inputs to the ORB primarily originate from the MD and SMT of the TH, with the perireunensis nucleus (PR) contributing a minor input ratio. Within the MTN, the paraventricular nucleus (PVT) and parataenial nucleus (PT) of the TH preferentially provided inputs to the ORBm, while the nucleus of reuniens (RE) provided the highest proportion of inputs to the ORBvl. The remaining thalamic subregions provided minimal inputs to the ORB subregions (Figure 7B). Our data further showed that the ipsilateral TH provided the majority of inputs to the ORB (Figure 7C), with sparse inputs originating from the contralateral TH (Supplementary Figure 7) and some contralateral thalamic subregions did not innervate ORB at all.

## Discussion

**FIGURE 8:**
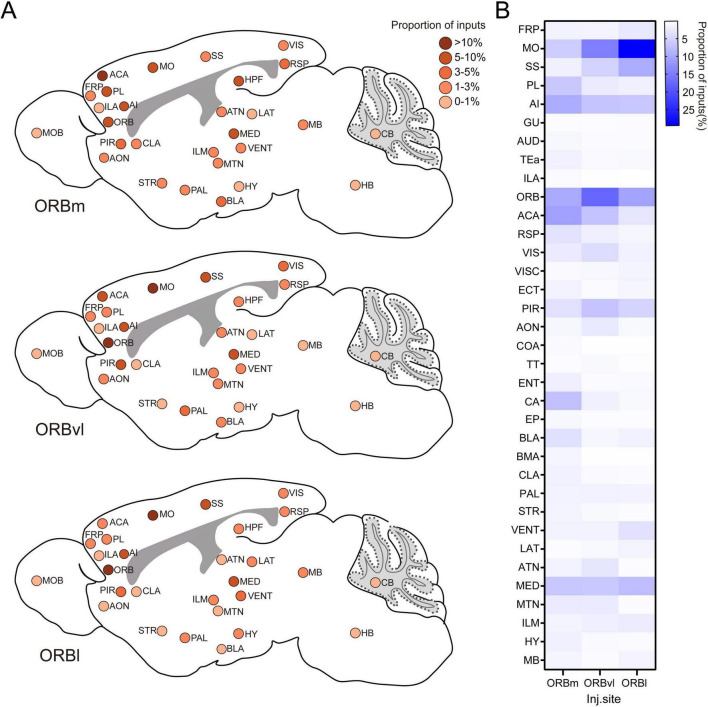
Whole-brain input patterns of the orbitofrontal cortex (ORB) subregions. **(A)** Schematic representation of whole-brain inputs to the ORB subregions. This illustration highlights the predominant input sources of the medial part (ORBm), the ventrolateral part (ORBvl), and the lateral part (ORBl). The input intensities from various brain regions are denoted by the different colors, reflecting the relative contributions of each brain region to the overall input patterns of the ORB. **(B)** Comparison of the input intensity of the ORB subregions. This graph summarizes the quantitative analysis of input cells from various brain regions to the ORBm, ORBvl, and ORBl.

In this study, we utilized a rabies virus-based method to create a comprehensive map of the whole-brain input distribution of ORB neurons. This approach allowed us to identify the distinct input patterns for each ORB subregion, as well as any differences in their whole-brain connectivity. Our research findings indicate that the upstream neurons providing input to the ORB stem from a variety of brain regions, encompassing the BLA, TH, OLF, gustatory areas, auditory areas, AI, and RSPagl (Figure 8). These findings align with earlier anterograde and retrograde tracing studies carried out in rodents (Kita and Kitai, 1990; Reep et al., 1996; Carmichael and Price, 1996; Price, 2007; Zingg et al., 2014; Xiang et al., 2023). Furthermore, our results provide a more detailed illustration of the differential proportions of inputs to the three ORB subregions from these brain areas. Notably, while the BLA predominantly innervates ORBm, thalamic inputs to ORBl clearly originate from the SMT, in contrast to the weak SMT projections to ORBm and ORBvl. Additionally, we have uncovered novel input pathways to the ORB, including robust projections from both the RSPd and RSPv to all three subregions, the minimal inputs from visceral area to all three ORB subregions, as well as inputs from the CA1 to ORBvl and ORBl. Most importantly, the primary contribution of our work lies in the quantitative analysis, comparison, and interpretation of input cells to the three ORB subregions across the entire brain (Figure 8), revealing the overall pattern of whole-brain inputs to the ORB: Ipsilateral brain regions provided more inputs; however, some contralateral regions also provided neural innervation to ORB. Our data also demonstrated that the ORB is composed of complex internal circuitry. For example, the ORB comprises abundant local projections, among which contralateral inputs are particularly prominent. While there is a general similarity in the input patterns across the ORB subregions, we identified notable variations in the proportional contribution of specific brain regions. For example, the ORBm received more inputs from regions, such as the PL, AI, and CA, compared to the ORBvl and ORBl. In contrast, the ORBvl received more inputs from within the ORB itself, whereas the ORBl exhibited a higher proportion of inputs from the MO, SS, and SMT. These patterns offer valuable insights into the respective roles of each subregion and provide robust support for existing functional studies.

It should be noted that some of our results are also inconsistent with previous findings. For example, while our data identified input neurons to the ORB across RSPagl, RSPd, and RSPv, anterograde tracing studies only show that the rodent homolog of posterior cingulate area 23 (approximating RSPagl) projects to the ORB (Xiang et al., 2023). Moreover, studies using the anterograde tracer Phaseolus vulgaris-leucoagglutinin have shown that the SUB in the HPF has extensive projections to the ORBm within the ORB, but no inputs to the ORBvl and ORBl (Jay and Witter, 1991). However, our results indicate that both ORBm and ORBvl receive extremely sparse inputs from the SUB, while ORBl receives no input from the SUB. Such discrepancies likely stem from methodological distinctions inherent in distinct labeling strategies. For instance, Cao’s lab demonstrated that rAAV2-retro preferentially labels cortical inputs to the lateral hypothalamic area and medial preoptic nucleus, whereas RV-ΔG favors subcortical regions like the basal ganglia and hypothalamus (Sun et al., 2019). In addition, Deisseroth’ lab utilized RV-tdTomato retrograde tracing and AAV5-eYFP anterograde labeling to identify ACA projections to the hippocampus region (HIP) (Rajasethupathy et al., 2015). In contrast, Zhuo’ lab employing AAV2/9 anterograde tracing, failed to detect ACA→HIP connectivity (Shi et al., 2022). These tropism differences may lead to incomplete or biased circuit mapping. To resolve such ambiguities, future studies should adopt multimodal validation strategies, combining intersectional genetic tools (e.g., Cre-dependent AAV/RV systems) with functional interrogation (optogenetics, pharmacogenetics). So, neuroanatomical conclusions must account for tracer-specific biases, and cross-methodological consistency remains paramount for robust circuit validation.

### Patterns of isocortical input to the ORB

Our study has shown that the isocortex projects extensively into the ORB, which is consistent with prior research (Reep et al., 1996; Ongür and Price, 2000). These projections are broadly distributed among the various regions of the isocortex. Known for its role in processing sensory information, the isocortex engages in several functions, including motor command generation and execution, sensory perception, cognitive processing, and emotional responses (Van Essen and Glasser, 2018; Felleman and Van Essen, 1991; Rakic, 2009). Our findings emphasize the vital importance of the ORB in the brain’s handling of these informational pathways and the performance of these diverse functions.

Our analysis further demonstrated the distribution of inputs from the isocortex to the ORB. The PL, AI, RSP, CA, and BLA were notable for providing preferential inputs to the ORBm. Considering the functions of these isocortical regions, their contribution to the ORBm hints at the subregion’s potentially pivotal role in emotional regulation and cognitive mapping. Conversely, ORBvl neurons received a greater share of inputs from within the ORB itself, suggesting its heightened involvement in intraregional interactions and possible role in complex emotional processing. Meanwhile, ORBl neurons received more inputs from the MO and SS, implying their integral role in motor control, sensory processing, and the modulation of learned behaviors.

These results underscore the distinct roles of the ORB subregions in mediating isocortex-driven functions. The varied input patterns from isocortex regions suggest specialized contributions of the ORBm, ORBvl, and ORBl to emotional regulation, motor control, and other cognitive processes. Further exploration of the functional interplay between these ORB subregions and their isocortical inputs is needed to fully decipher their roles in brain function.

### Input to the ORBm from the isocortex

All three ORB subregions received considerable inputs from the AI, with the ORBm receiving particularly strong inputs. The AI and ORBm are closely associated with emotional regulation; the AI deals with emotional responses (Nieuwenhuys, 2012; Butti and Hof, 2010), while the ORBm specializes in emotional processing (Rolls, 2019; O’Doherty et al., 2003). Previous studies have established a bidirectional relationship between all three AI subregions and the ORB (Gehrlach et al., 2020). While they did not study the projection weights between these ORB subregions, our research provides valuable structural insights into their interconnections. It also raises the intriguing question of whether this bidirectional projection forms a closed loop for integrating and regulating information between the AI and ORB. Understanding this loop is crucial in further understanding the complex roles of AI and ORB pathways in emotional regulation mechanisms. Advanced methodologies like optogenetics may be instrumental in verifying their functional contributions.

### Input to the ORBvl from the isocortex

All three ORB subregions received robust inputs from the ORB itself. This may be evidence of self-regulation or a mechanism by which the ORB can maintain functional stability and adaptability in complex, dynamic environments. Notably, the ORBvl received substantially more inputs from the ORB than the ORBm and ORBl, highlighting its key role in integrating internal ORB information. In decision-making scenarios, the ORBvl relays choice-related signals to the dorsomedial STR, which contributes to decision-making (Gore et al., 2023). Furthermore, the ORB-ORBvl pathway may integrate rewards and emotional inputs from various ORB subregions to modulate dorsomedial STR activity. The ORBvl also exhibited strong innervation from the contralateral ORBvl and ORBl. This interhemispheric connection aids in synchronizing and integrating information during complex cognitive and emotional processing. The ORBl has been shown to participate in processing punishment information and its activation levels correlate with punishment severity (O’Doherty et al., 2001). Inputs from the contralateral ORBvl and ORBl may enrich the ORBvl’s capacity for punishment processing and decision-making.

Structural and functional abnormalities within the ORB have been documented in patients with depression. Patients in depressive states exhibit more functional connectivity between the right inferior frontal gyrus and the ORB (Rolls et al., 2020). Adults diagnosed with depression demonstrate increased cortical thinning within the gray matter of the ORB (Yang and Kwan, 2021). Adolescents with depression exhibit a similar decrease in total surface area (Yang and Kwan, 2021). Additionally, patients with depression exhibit increased functional connectivity in the ORBl but decreased connectivity in the ORBm (Rolls et al., 2018). Recent research supports a connection between the ORBvl and depression, with studies demonstrating the area’s involvement in depressive behaviors and the response to anti-depressants like valproic acid, a mood stabilizer (Xing et al., 2011). Other studies have also shown that impairment of the ORBvl is associated with a reduction in depressive behavior (Chu et al., 2021). All these contribute to highlighting the ORB as a promising therapeutic target for depression. Ultimately, our data showed that the ORBvl receives extensive projections from the other ORB subregions, particularly the contralateral ORBvl. Elucidating how these projections contribute to the pathogenesis of depression may improve our understanding of the disease, as well as propose novel treatment strategies.

### Input to the ORBl from the isocortex

The ORBm, ORBvl, and ORBl all received substantial inputs from the MO, with the ORBl receiving significantly more inputs than the ORBm and ORBvl. The MOs are primarily responsible for associating prior decisions and sensory stimuli with upcoming motor actions (Yang and Kwan, 2021). Given the central role of the ORBl in processing punishment information, the MO-ORBl pathway may similarly participate in intricate cognitive-motor integration processes by (1) receiving sensory and motor information from the MOs and (2) integrating this information with other internal signals from the ORB to generate precise and comprehensive cognitive information. This information may serve as a guide for performing appropriate motor actions based on past experiences and decisions.

The present study identified similar input patterns between the SS and MO and the ORB subregions. Both brain regions provided preferential inputs to the ORBl, followed by the ORBvl and ORBm. Further analysis revealed that subregions of the SS, the SSp and the SSs, provided significantly more inputs to the ORBl compared to the ORBm, with the SSp providing the majority of these inputs. Prior research has indicated that the ORBl-SSp pathway may regulate value prediction, which can contribute to flexible decision-making (Banerjee et al., 2020). The data from our study further suggest that there are bidirectional projections between the ORBl and SSp. This may lay the neural groundwork for value prediction, which can help integrate somatosensory information with emotional and cognitive processes to guide behavior.

### Patterns of hippocampal input to the ORB

Our research has also revealed that the CA region of the hippocampus projects to the ORB, specifically the ORBm. Over 90% of the CA neurons participating in this connection originate from the CA1. Previous studies have shown that CA neurons are crucial for learning, memory, and spatial navigation (Rolls, 2022; Chowdhury et al., 2022). The studies have demonstrated a connection between the ORB and hippocampus, which allows the ORB to convey rewards information to the hippocampus, thereby facilitating episodic memory function (Rolls, 2022). The potential role of the CA-ORBm pathway in transmitting rewards information and integrating episodic memory is a good topic for future study.

### Patterns of thalamic input to the ORB

Our findings demonstrate that thalamic inputs to the ORB predominantly originate from MD, posterior complex of the thalamus (PO), SMT, PVT, ventral medial nucleus of the thalamus (VM), RE and rhomboid nucleus (RH), which aligns with prior anatomical studies (Reep et al., 1996; Barreiros et al., 2021; Zhang et al., 2024). Specifically, MD and SMT exhibited robust projections to all three ORB subregions (ORBm, ORBvl, ORBl), while PVT, VM, RH, and RE provided comparatively weaker inputs.

Previous work reported that the posterior lateral ORB receives inputs from parataenial nucleus (PT) and RE, while the posterior ventral ORB is innervated by RE (Barreiros et al., 2021). Our results further revealed minor PT projections to ORBl and ORBvl, expanding current knowledge of thalamo-ORB connectivity.

Our quantitative comparisons of thalamic subregional input preferences revealed that MD contributed the highest proportion of thalamic inputs to the ORB, with no significant differences in input intensity across the three ORB subregions. MD is critically implicated in advanced cognitive functions, including cognitive control (DeNicola et al., 2020), working memory (Watanabe and Funahashi, 2012; Oyama et al., 2021), and decision-making (Ding et al., 2024). Intriguingly, silencing the ORB-MD pathway impairs inference-based value updating (Oyama et al., 2024), raising critical questions: What roles do MD-to-ORB projections play in MD-mediated cognitive processes such as working memory and decision-making? Given the bidirectional connectivity between ORB and MD (Oyama et al., 2024), how do these reciprocal circuits orchestrate higher-order cognitive functions? Future studies should address these gaps.

We also observed distinct thalamic input patterns: SMT exhibited stronger inputs to all ORB subregions compared to PO and PVT, while the ventral anterior-lateral complex (VAL) and SMT preferentially innervated ORBl. In contrast, PVT and PT displayed biased projections to ORBm. Although the anteromedial nucleus (AM) and RE provided modest overall inputs to the ORB, both regions exhibited a consistent hierarchy of innervation—highest to ORBvl, followed by ORBm and ORBl—with AM and RE inputs to ORBvl significantly exceeding those to ORBl. Functional implications of these patterns warrant further exploration. PO, SMT, and PVT are implicated in nociceptive modulation (Wang H. et al., 2020; Tang et al., 2009; Liu et al., 2025; Li et al., 2024), while AM and RE regulate fear memory (Ramanathan and Maren, 2019; Troyner and Bertoglio, 2021; Lv et al., 2021). The thalamo-ORB pathways identified here may serve as anatomical substrates for integrating nociceptive or fear-related signals with ORB-mediated decision-making and emotional processing. Elucidating these functional interactions will deepen our understanding of ORB-TH circuitry in both health and disease.

Future research should delve deeper into the functional implications of the input patterns to each ORB subregion. In particular, future efforts may explore how these inputs contribute to value prediction, decision-making, and the integration of somatosensory, emotional, and cognitive processes.

Our study focused exclusively on male mice, a design choice that inherently limits insights into potential sex-dependent circuit organization of the ORB. This limitation is particularly salient given emerging evidence for functional and structural sexual dimorphism in the ORB. For instance, recent studies demonstrate that cocaine self-administration induces sex-specific alterations in synaptic properties of parvalbumin interneurons within the ORB (Wright et al., 2021). Additionally, female rats exhibit slower acquisition but more robust, stress-resistant contextual control over rewards-seeking behavior, accompanied by heightened ORB activation—a finding that highlights sex-divergent neural mechanisms potentially linked to psychiatric vulnerabilities (Peterson et al., 2024). These observations raise fundamental questions about whether sex differences in ORB-dependent behaviors arise from structural or functional disparities in its connectivity. While our current work establishes a foundational map of ORB subregion connectivity in males, systematic comparisons of whole-brain input-output patterns across sexes will be essential to unravel the circuit basis of these functional divergences.

## Data Availability

The original contributions presented in this study are included in this article/Supplementary material, further inquiries can be directed to the corresponding author.
