# Supplementary material for: Anatomical mapping of whole-brain monosynaptic inputs to the orbitofrontal cortex
Source: Front Neural Circuits. 2025 Apr 4;19:1567036. doi: 10.3389/fncir.2025.1567036 (PMC12006047; doi:10.3389/fncir.2025.1567036)
Supplement: Supplementary file 1 [file Data_Sheet_1.docx]

Supplementary Material


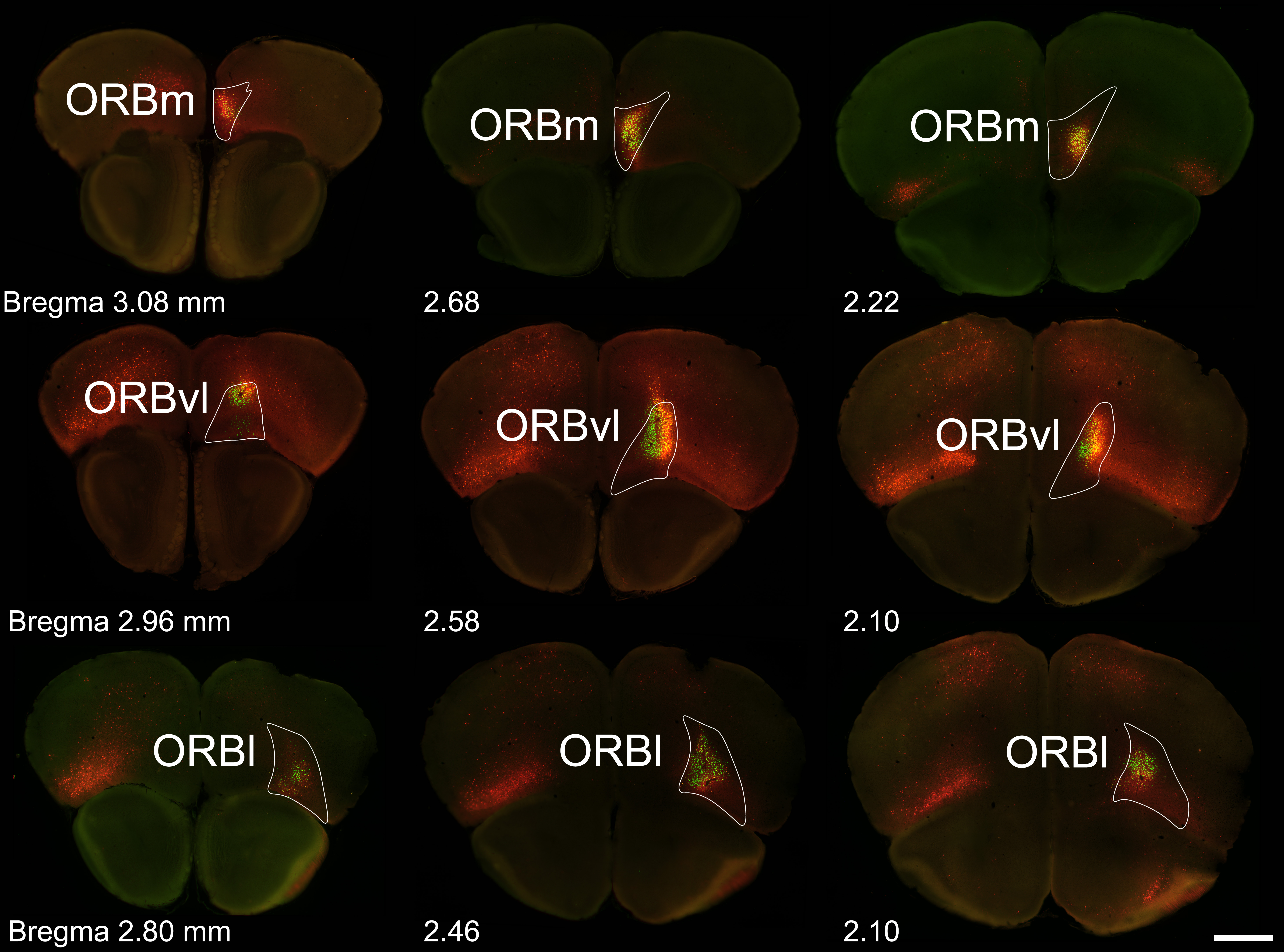


**Supplemental Figure S1.** Representative imaging of viral injection sites in ORB subregions. EGFP fluorescence (green channel) confirms targeted viral delivery in the ORBm (top row), ORBvl (middle row), and ORBl (bottom row). Scale bars: 1000 µm.

**
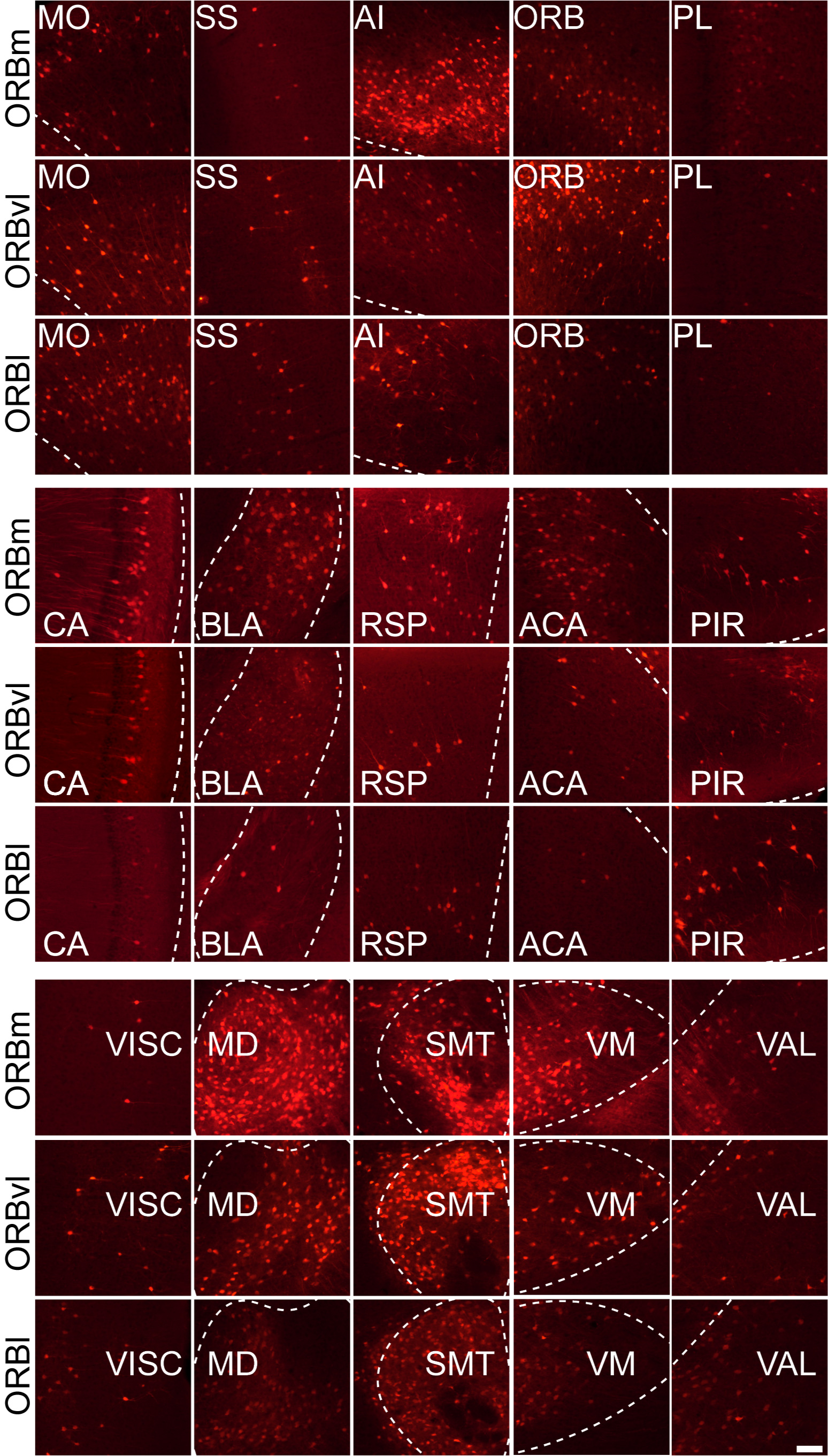
**

**Supplemental Figure S2.** Representative images of multiple input brain regions projecting to the ORB subregions. This figure shows representative images of multiple brain regions located on the same side as the injection site. In one particular case, a representative image of the contralateral ORB is shown (Scale bar, 100 μm).

**
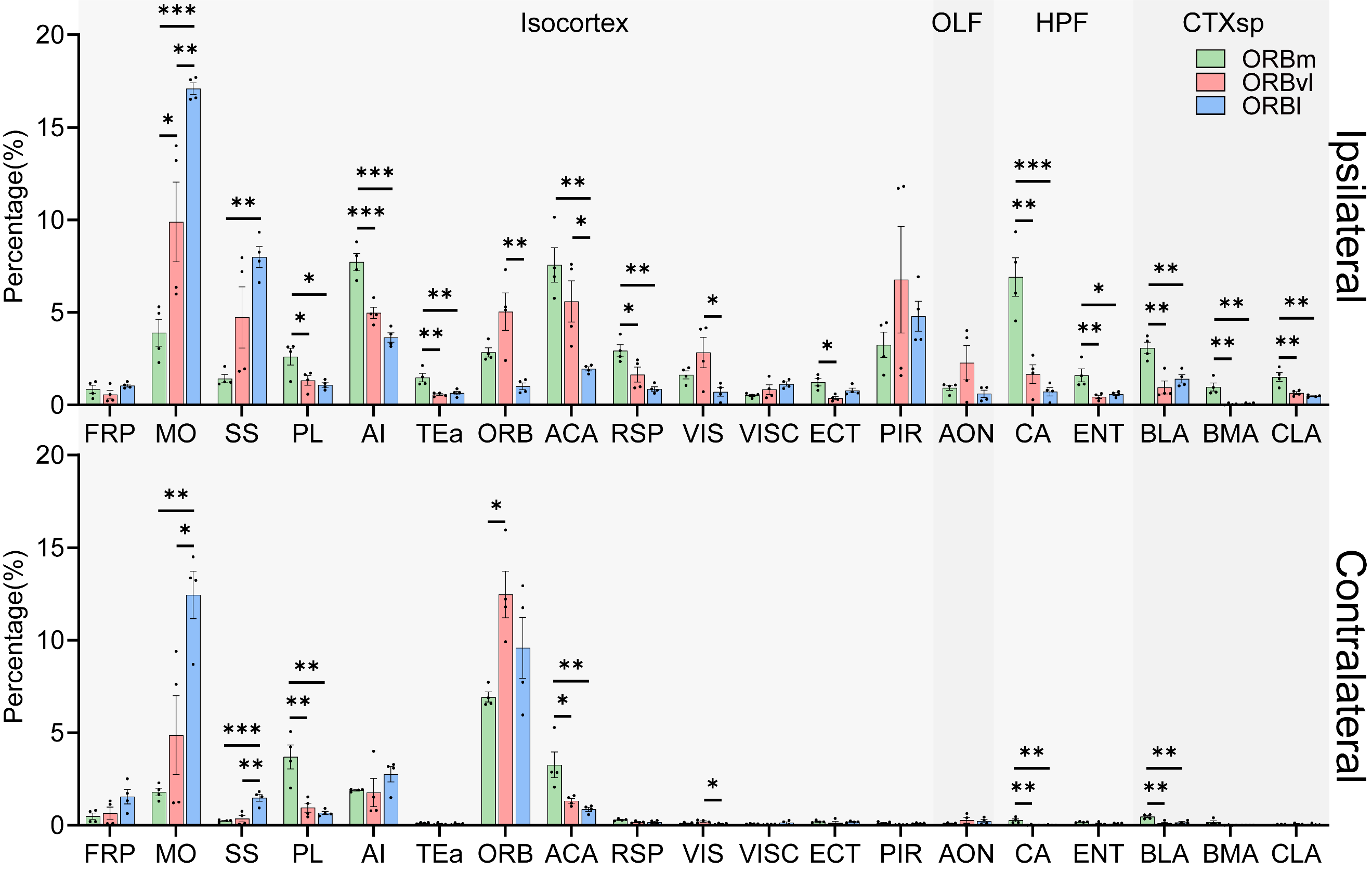
**

**Supplemental Figure S3.** Distribution of input cells projecting to the ORB subregions in the ipsilateral and contralateral CTX. **P* < 0.05, ***P* < 0.01, ****P* < 0.001. Data are presented as the Mean ± SEM (n = 4 mice/group). One-way ANOVA combined with Tukey’s multiple comparisons test.


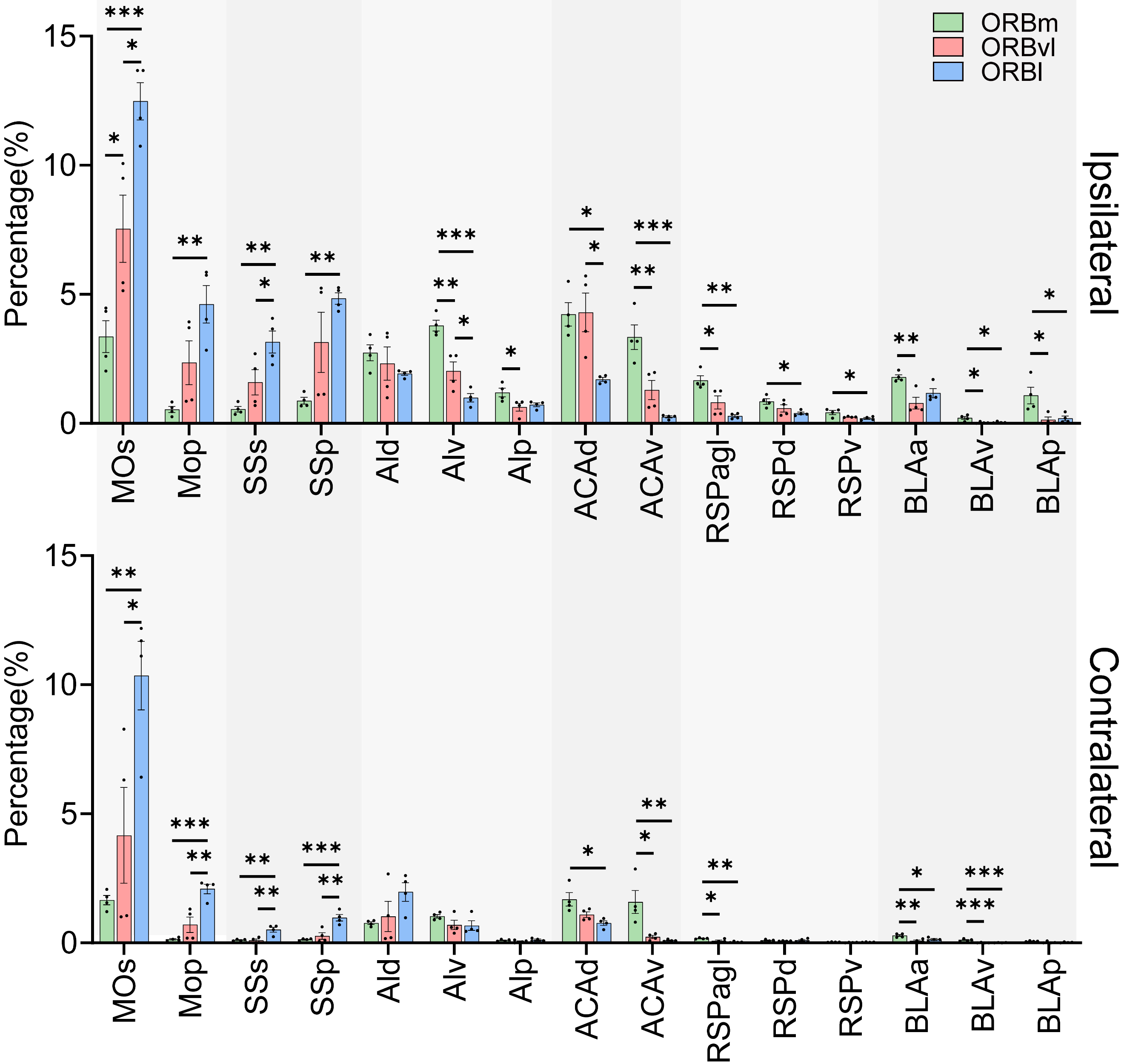


**Supplemental Figure S4.** Distribution of input cells from the ipsilateral and contralateral MO, SS, AI, ACA, RSP, and BLA projecting to the ORB subregions. **P* < 0.05, ***P* < 0.01, ****P* < 0.001. Data are presented as the Mean ± SEM (n = 4 mice/group). One-way repeated measures ANOVA followed by Tukey’s multiple comparisons test.

**
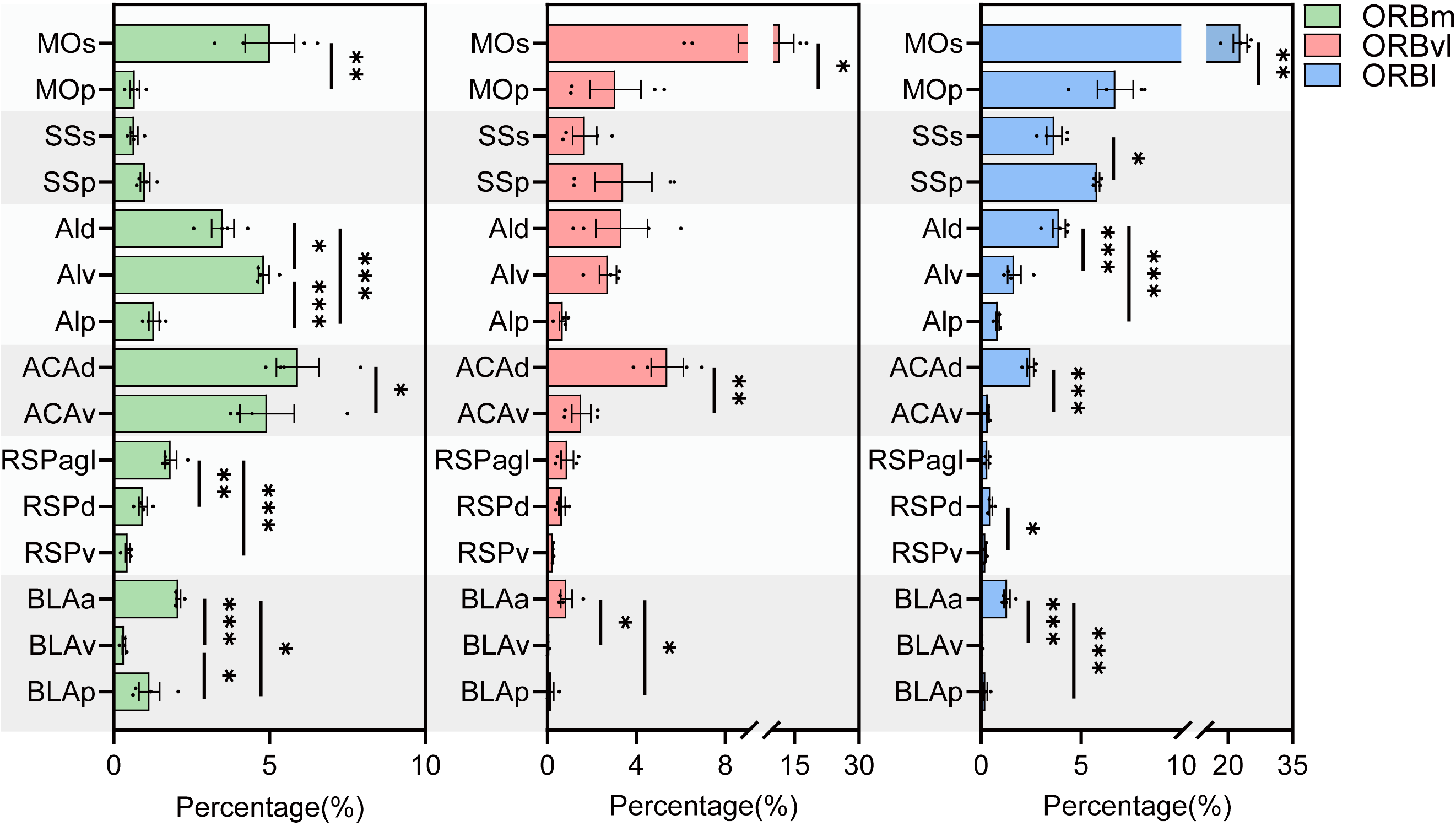
**

**Supplemental Figure S5.** Distribution of input cells from the MO, SS, AI, ACA, RSP, and BLA projecting to each ORB subregion. **P* < 0.05, ***P* < 0.01, ****P* < 0.001. Data are presented as the Mean ± SEM (n = 4 mice/group). The Student’s *t*-test was used for samples with two data sets. One-way repeated ANOVA followed by Tukey’s multiple comparisons test was used for samples with three data sets.


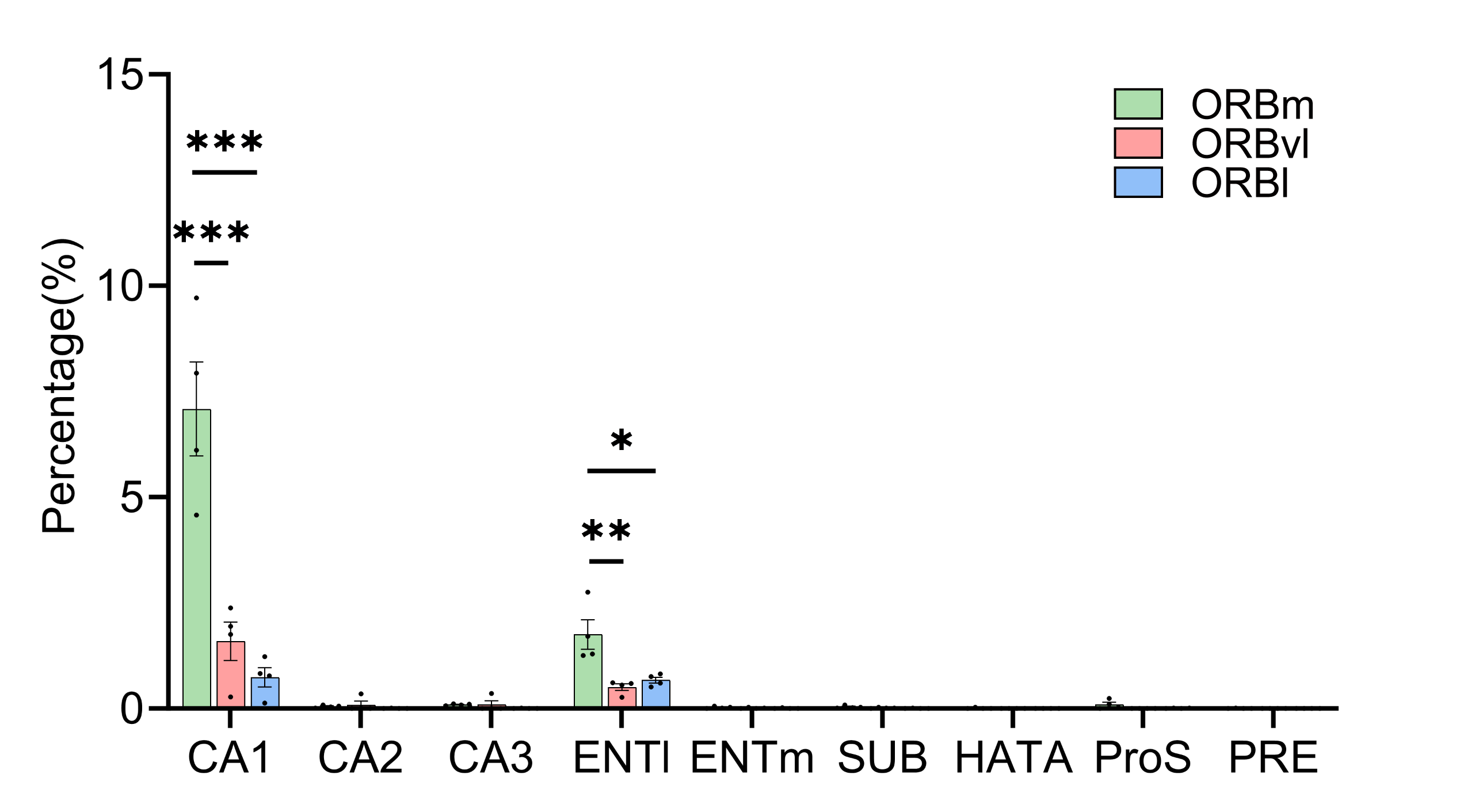


**Supplemental Figure S6.** Distribution of input cells from the HPF projecting to the ORB subregions. **P* < 0.05, ***P* < 0.01, ****P* < 0.001. Data are presented as the Mean ± SEM (n = 4 mice/group). One-way ANOVA combined with Tukey’s multiple comparisons test.


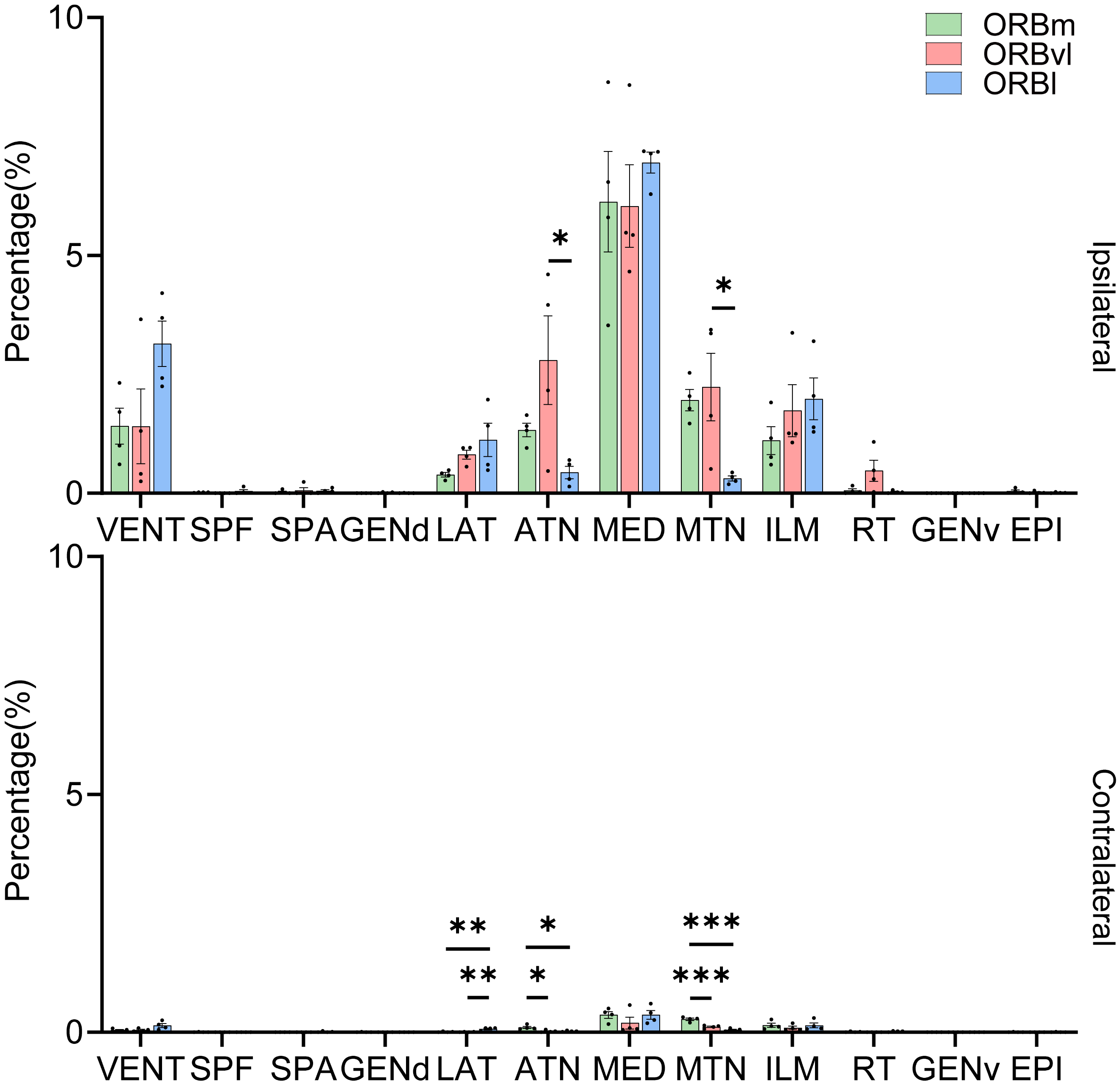


**Supplemental Figure S7.** Distribution of input cells from the ipsilateral and contralateral TH projecting to the ORB subregions. **P* < 0.05, ***P* < 0.01, ****P* < 0.001. Data are presented as the Mean ± SEM (n = 4 mice/group). One-way ANOVA combined with Tukey’s multiple comparisons test.
